# Spontaneously Ruptured Gastrointestinal Stromal Tumor With Pelvic Abscess: A Case Report and Review

**DOI:** 10.4021/gr2009.11.1324

**Published:** 2009-11-20

**Authors:** Serife Ulusan, Zafer Koc, Fazilet Kayaselcuk

**Affiliations:** a Baskent University, Faculty of Medicine, Dept.of Radiology, Adana Teaching and Medical Research Center, Turkey; b Baskent University, Faculty of Medicine, Dept. of Pathology, Adana Teaching and Medical Research Center, Turkey

**Keywords:** Gastrointestinal stromal tumors, X-Ray computed tomography, Abdominal abscess

## Abstract

Gastrointestinal stromal tumors (GISTs) originate from interstitial Cajal cells on intestinal pacemaker cells that arise from the muscularis propria of the gastrointestinal tract wall. GISTs are characterized by the expression of c-KIT protein (CD 117, stem cell factor receptor) and are the most common mesenchymal tumors of the digestive tract. That protein, which is detected via immunohistochemical analysis, is the primary diagnostic criterion for a GIST. The rupture of a gastrointestinal stromal tumor of the peritoneal cavity is critical complication, although it is infrequently described in the literature. We describe the computed tomographic findings of a ruptured gastrointestinal stromal tumor of the jejunal wall with an accompanying abscess. We also review the clinical features, radiologic and pathologic findings, and treatment of similar previously reported cases.

## Introduction

Gastrointestinal stromal tumors (GISTs), which are the most common ‘mesenchymal’ tumor of the gastrointestinal tract, are nonepithelial neoplasms that arise from the muscularis propria of the gastrointestinal tract wall. GISTs originate from interstitial Cajal cells on intestinal pacemaker cells [1, 2]. GISTs have been classified as spindle cell, epithelioid, or (occasionally) pleomorphic mesenchymal tumors of the gastrointestinal tract that express the KIT protein (CD 117, stem cell factor receptor). That protein, which is detected via immunohistochemical analysis, is the primary diagnostic criterion for a GIST [1, 2].

Tumors can develop anywhere in the gastrointestinal tract. GISTs occur most frequently in the stomach (60%) and then most often in the small bowel (30%); other sites include the colon and rectum (5%) and the esophagus (< 5%) [1-4]. GISTs account for 1% to 3% of gastric neoplasms, 20% of small bowel tumors, and 0.2% to 1% of colorectal tumors [1-4].

Although there are a few reports of ruptured GISTs, this is to our knowledge the first case in the English literature of a GIST with an accompanying abscess.

## Case Report

A 52-year-old woman with abdominal pain and tenderness, fever, and swelling of the lower abdomen of a few days’ duration was admitted to our institution. Physical examination revealed a palpable abdominal mass.

**Figure 1:**
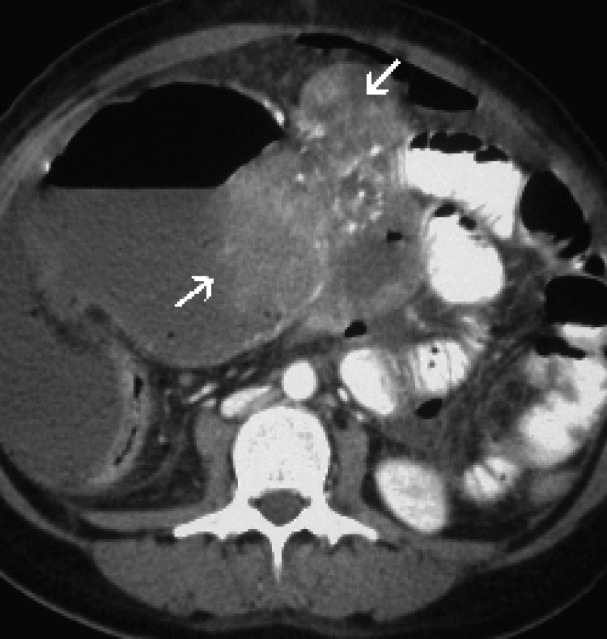
A postcontrast computed tomographic scan showed a huge primary calcified GIST (arrows) with a cystic component.

**Figure 2:**
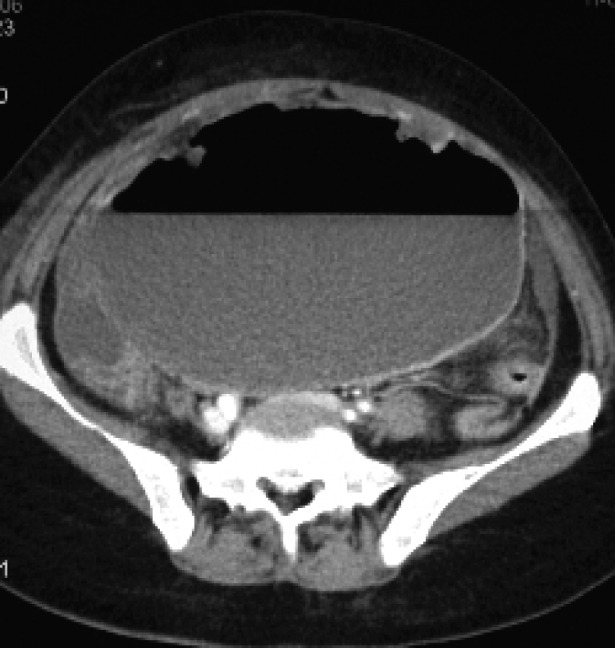
A postcontrast computed tomographic scan revealed a pelvic abscess with an air-fluid level.

A preoperative computed tomographic scan showed a huge primary calcified soft tissue tumor (18 x 13 x 7 cm) in the upper abdomen. A cystic component of the tumor and hemoperitoneum were also identified (Fig. 1). One cystic lesion (10 x 11 x 8 cm) with an air-fluid level was detected in the pelvic region (Fig. 2).

The findings at surgery confirmed that the primary GIST site was identified from the preoperative computed tomographic scan. The primary tumor had ruptured on 2 sides, and what appeared to be a cystic pelvic lesion on computed tomographic examination was a large pelvic abscess. Culture of the abscess yielded Enterococcus species.

**Figure 3:**
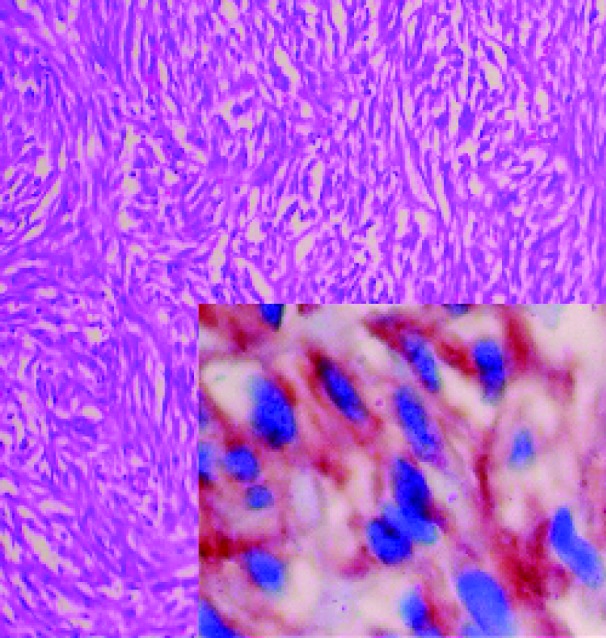
On microscopic examination the tumor showed spindle cells with round hyperchromatic nuclei and prominent nucleoli forming bundles (haemotoxylin and eosin); 200 x Inset: Immunohistochemically, the cells were diffusely positive for CD-117 (c kit), CD-117, 200 x.

The results of pathologic examination suggested that the primary lesion had originated in the serosa of the small bowel and had not invaded adjacent visceral organs. The mitotic index of the lesion was 3 mitoses per 50 high-power fields (HPFs). The tumor formed by spindle cells with round hyperchromatic nuclei and prominent nucleoli forming bundles immunohistochemically; the cells were diffusely positive for CD-117 (c kit) and focal and weak positivite for S-100; SMA, CD-34 and desmin were negative (Fig. 3). Chemotherapy with imatinib (Gleevec, Novartis, Switzerland) was initiated because of the large size of the tumor (> 5 cm), the presence of coagulative tumor necrosis, borderline mitotic activity (3/50 HPFs), and confirmed rupture of the neoplasm. Our patient gave written informed consent to radiologic examination and to participation.

## Discussion

In the gastrointestinal tract, CD 117-positive cells (interstitial Cajal cells) are autonomic nerve-related gastrointestinal pacemaker cells that regulate intestinal motility [1, 2]. Because of the immunohistochemical and ultrastructural similarities of Cajal cells and GISTs, a histogenetic origin of GISTs from Cajal cells has been proposed [1, 2].

A few previous studies have investigated ruptured GISTs by means of several radiologic modalities such as ultrasonography and computed tomography. A literature search yielded only 9 reports of ruptured primary GISTs to date. Cegarra-Navarro et al reported 5 patients with a ruptured primary GIST and 1 patient with a ruptured metastatic GIST [5]. We did not evaluate ruptured metastatic GISTs. One of the nine patients’ tumours was small bowel location and another one was transverse mesocolon location; the others were gastric location [5-9]. Seven of the nine patients’ ruptured GISTs were spontaneous [5-9]; the others were result of the trauma [5].

To our knowledge, this case is the second published report of a spontaneously ruptured GIST in the small bowel. Eight cases of a GIST with hemoperitoneum and 1 case of a GIST with concomitant hemoperitoneum and peritonitis have been reported [5, 7-9]. Our patient demonstrated a GIST with hemoperitoneum and abscess but without peritonitis.

The computed tomographic findings of ruptured GISTs (noncontrast and postcontrast images) showed huge masses of low density with exophytic growth in the peritoneal cavity and ascites. These ruptured tumors are echogenic on ultrasonography and dense on computed tomographic scans (findings that suggest hemoperitoneum). The size of 3 of the 9 previously reported GISTs was less than 10 cm [5], and the 6 remaining GISTs were larger than 10 cm [5-8]. As in most of the other cases reported, the mass found in our patient was larger than 10 cm.

Seven of the previously reported GISTs exhibited low mitotic activity (less than 5 mitoses per 50 HPF) [5-9]. Two of the reported ruptured primary GISTs demonstrated a high mitotic index (20 mitoses per 50 HPF) [5-9]. As in most of the other cases reported, our patient’s tumor was characterized by borderline mitotic activity.

All primary GISTs that were previously reported except 2 demonstrated low or borderline mitotic activity [5-8], but all had a large cystic and necrotic component. We may speculate that the rupture of primary GISTs involves the cystic and necrotic tumor components but not high-grade mitotic activity.

Most of the patients with a ruptured GIST presented for urgent care and all patients underwent emergency laparotomy [5-9]. Segmental small bowel or gastric resection is standard treatment for perforated local GIST. Imatinib is being evaluated as adjuvant treatment following surgery, especially in high malignant potential cases [10].

To our knowledge, a ruptured GIST with an accompanying abscess has not been previously reported. We describe the postcontrast computed tomographic findings, surgical correction, and histopathologic findings of a ruptured primary GIST and review the clinical and radiologic follow-up. In our patient, the operation was successful, and she did well after surgery.
